# Normal/high-fat milk consumption is associated with higher lean body and muscle mass in Japanese women aged between 40 and 60 years: a cross-sectional study

**DOI:** 10.1186/s12905-018-0525-0

**Published:** 2018-02-02

**Authors:** Yuri Sukenobe, Masakazu Terauchi, Asuka Hirose, Miho Hirano, Mihoko Akiyoshi, Kiyoko Kato, Naoyuki Miyasaka

**Affiliations:** 1 Departmant of Obstetrics and Gynecology, Tokyo Medical and Dental University, Yushima 1-5-45, Bunkyo, Tokyo, 113-8510 Japan; 2 Departmant of Women’s Health, Tokyo Medical and Dental University, Yushima 1-5-45, Bunkyo, Tokyo, 113-8510 Japan

**Keywords:** Low-fat milk, Body composition, Cholecalciferol

## Abstract

**Background:**

Milk is known to contain various nutrients that may have health benefits for postmenopausal women who are at an increased risk of cardiovascular and musculoskeletal diseases. We investigated the association between normal/high- and low-fat milk consumption and body composition in Japanese women aged 40 to 60 years.

**Methods:**

This cross-sectional study used the baseline data collected in a previous study that examined the effects of a dietary supplement on a variety of health parameters in 85 Japanese women aged 40 to 60 years. Participants had been assessed for age, menopausal status, lifestyle factors, and body composition. We estimated the consumption of normal/high- and low-fat milk using a brief-type self-administered diet history questionnaire (BDHQ). Normal/high- and low-fat milk intake were classified as consumer (drank milk at least twice a week) or non-consumer (drank milk at most once a week), in order to identify the parameters that were independently associated with the consumption of normal/high- and low-fat milk.

**Results:**

Of the 85 participants who completed the BDHQ, 27 were categorized as non-consumers, 18 as exclusive low-fat milk consumers, and 29 as exclusive normal/high-fat milk consumers. 11 women who consumed both low-fat and normal/high-fat milk were excluded from the analysis. Compared with non-consumers and exclusive low-fat milk consumers, exclusive high-fat milk consumers had significantly higher lean body mass (mean ± standard deviation [SD], 39.4 ± 2.7 kg vs. 37.9 ± 2.2 kg and 37.6 ± 2.9 kg, *P* < 0.05) and muscle mass (mean ± SD, 37.2 ± 2.5 kg vs. 35.8 ± 2.0 kg and 35.5 ± 2.7 kg, *P* < 0.05). Both lean body and muscle masses were significantly correlated with vitamin D intake from milk (Pearson *r* = 0.29, *P* = 0.008, and Pearson *r* = 0.29, *P* = 0.008, respectively).

**Conclusion:**

Normal/high-fat milk consumption was associated with higher lean body and muscle mass in middle-aged Japanese women presumably through high vitamin D intake.

## Background

Menopause is defined as the permanent cessation of menstruation resulting from the loss of ovarian follicular activity, characterized by decreased serum estrogen levels [1]. Postmenopausal women are vulnerable to cardiovascular and musculoskeletal diseases such as myocardial infarction, osteoporosis and sarcopenia, which are partly attributable to the changes in body composition induced by the loss of estrogen [2–4]. For example, abdominal obesity increases the risk of cardiovascular diseases (CVD) [5, 6], whereas low muscle mass increases the risk of falls and insulin resistance [7].

It has been shown that appropriate exercise and nutrition is crucial for the prevention of chronic diseases in middle aged women [8]. Milk is known to contain various nutrients, such as minerals, vitamins, proteins, and essential amino acids, which support osteogenesis, lipid metabolism, and myogenesis. Calcium, a mineral that is not absorbed sufficiently by most Japanese children and adults due to lactose malabsorption [9], and vitamin D, which maintains homeostasis of intracellular calcium and phosphorus [10], are essential for osteogenesis. Additionally, vitamin B2 promotes lipid metabolism [11] . Milk is also rich in branched-chain amino acids (BCAA), which suppress protein degradation and facilitates the protein synthesis of muscles [12]. Recently, high dose of milk product intake after exercise was reported to increase thigh muscle strength possibly through NFKB1 and NFKB2 gene methylation in elderly women [13]. However, little is known about the association between daily milk consumption and the body composition in middle-aged women.

In the present study, we investigated the association between milk intake and body composition in Japanese women aged 40 to 60 years.

## Methods

### Study population

We performed a cross-sectional analysis using the baseline data from a previous study conducted at the Menopause Clinic of Tokyo Medical and Dental University from November, 2012 to June, 2013 that examined the effect of a dietary supplement on a variety of health parameters in 85 Japanese women [14]. Women were included if they were between 40 and 60 years of age and had at least one menopausal symptom scoring at least one on the Menopausal Health-Related Quality of Life (MHR-QOL) Questionnaire. Women were excluded if they were using menopausal hormone therapy, herbal medicine, psychotropic drugs, or dietary supplements at the time of recruitment. The participants were recruited through advertisements posted in our hospital and in the patients’ own social networks. Data collected included age, menopausal status, lifestyle factors, body composition, and dietary habits.

### Menopausal status

Women were defined as “premenopausal” if they had regular menstrual cycles in the past 3 months; as “perimenopausal” if they had a menstrual period within the past 12 months but had a missed period or irregular cycle in the past 3 months; as “postmenopausal” if they had no menstrual period in the past 12 months; and as “surgically induced menopause” if they had hysterectomy.

### Body composition

Body composition, including weight, body mass index, lean body mass, body water, estimated bone mass, visceral fat level, basal metabolic rate, muscle mass, fat mass, and fat percentage was assessed using a tetrapolar bioimpedance body composition analyzer (MC190-EM; Tanita, Tokyo, Japan). Anthropometric measurements and body composition estimates were done in the morning, after the study participants emptied their stomach, urinated, defecated, and performed stretching exercises. They had not been administered any diuretic medicine or/and liquids including caffeine 24 h before the body composition estimates. The lean body mass is calculated as body weight minus fat mass, and it includes the weight of organs, skin, bones, water, and muscles. The measurement error of the system was reported as less than 0.5 kg.

### Dietary habits

Dietary habits were assessed using a brief-type self-administered diet history questionnaire (BDHQ), a short version of a self-administered diet history questionnaire, which was developed in Japan [15]. The BDHQ inquired about the frequency of consumption of selected food and beverage items on 7-point scales, mainly from the food list used in the National Health and Nutrition Survey of Japan, which are commonly consumed in Japan. Based on the provided responses to the BDHQ, an ad hoc computer algorithm estimated the amounts of 98 nutritional factors consumed during the previous month. Table 1 shows the major nutritional factors assessed with BDHQ. Concerning normal/high- and low-fat milk, the scale of intake frequency is: twice a day or more (7); once a day (6); 4 to 6 times a week (5); 2 to 3 times a week (4); once a week (3); less than once a week (2); none (1). According to the participants’ responses, they were classified as “consumers” if they scored 4 or more and as “non-consumers” if they scored 3 or less. In the current study, the participants were categorized as: (1) “non-consumers”, who consumed neither low-fat nor normal/high-fat milk; (2) “exclusive low-fat milk consumers”, who consumed low-fat milk but not normal/high-fat milk; and (3) “exclusive normal/high-fat milk consumers”, who consumed normal/high-fat milk but not low-fat milk.

**Table 1:** Major nutritional factors assessed with BDHQ

Energy	Copper	Cholesterol
Weight of foods	Manganese	Soluble dietary fiber
Water	Retinol	Insoluble dietary fiber
Protein	Vitamin D	Dietary fiber
Animal protein	α-Tocopherol	Salt equivalent
Vegetable protein	Vitamin K	Sucrose
Fat	Vitamin B1	Alcohol
Animal fat	Vitamin B2	Daidzein
Vegetable fat	Niacin	Genistein
Carbohydrate	Vitamin B6	n-3 fatty acid
Ash content	Vitamin B12	n-6 fatty acid
Sodium	Folic acid	α-Carotene
Potassium	Pantothenic acid	β-Carotene
Calcium	Vitamin C	Cryptoxanthin
Magnesium	Saturated fatty acid	β-Tocopherol
Phosphorus	Monounsaturated fatty acid	γ-Tocopherol
Iron	Polyunsaturated fatty acid	δ-Tocopherol
Zinc		

*BDHQ* brief-type self-administered diet history questionnaire

### Study ethics approval

The study protocol was reviewed and approved by the Tokyo Medical and Dental University Review Board, and a written informed consent form was obtained from all participants. The study was conducted in accordance with the Declaration of Helsinki.

### Statistical analysis

Statistical analyses were performed using GraphPad Prism version 5.02 (GraphPad Software, San Diego, CA, USA). Univariate analyses, including unpaired t tests, chi squared (χ2) tests, and Mann-Whitney U tests, were conducted to test for differences between non-consumers, exclusive low-fat milk consumers, and exclusive normal/high-fat milk consumers concerning age, menopausal status, life style factors, and body composition. Additionally, we investigated the association between vitamin D intake from milk and lean body and muscle masses using regression analysis. *P* values < 0.05 were considered statistically significant.

## Results

Of the 85 participants, 27 were categorized as non-consumers, 18 as exclusive low-fat milk consumers, and 29 as exclusive normal/high-fat milk consumers (Table 2). Eleven women who consumed both low-fat and normal/high-fat milk were excluded from the analysis. The average BDHQ scores for low-fat and normal/high-fat milk consumption in each group were: 1.3 ± 0.7, 5.6 ± 1.0, 1.0 ± 0.2 and 1.9 ± 0.8, 1.2 ± 0.6, 5.9 ± 0.7, respectively (mean ± SD). The content of lean body mass, body fat, and body water were generally appropriate for the subjects’ age and sex. The estimated average daily intake of protein was not significantly different among non-consumers (59.7 ± 21.2 g), exclusive low-fat milk consumers (66.2 ± 16.1 g), and exclusive normal/high-fat milk consumers (62.3 ± 18.0 g) (mean ± SD, unpaired t test).

**Table 2:** Participant characteristics stratified by frequency of milk consumption

	Non- consumer	Exclusive low-fat milk consumer	Exclusive normal/high-fat milk consumer
	(*N* = 27)	(*N* = 18)	(*N* = 29)
Age, y	48.6 (5.7)	51.0 (5.1)	50.0 (4.8)
Menopausal status, %			
Premenopausal	44.4	38.9	51.7
Perimenopausal	22.2	11.1	13.8
Postmenopausal	33.3	38.9	24.1
Surgically induced menopause	0.0	11.1	10.3
Lifestyle factors, %			
Working	96.3	88.9	89.7
Exercising regularly	33.3	55.6	41.4
Smoking	11.1	0.0	13.8
Alcohol consumption			
(Daily	22.2	11.1	13.8
On occasion	51.9	66.7	55.2
Never)	25.9	22.2	31.0
Body composition			
Weight, kg	53.2 (7.0)	51.7 (7.5)	54.1 (6.8)
Body mass index, kg/cm^2^	21.9 (2.7)	21.5 (3.3)	21.8 (2.5)
Lean body mass, kg	37.9 (2.2)	37.6 (2.9)	39.4 (2.7)^ab^
Body water, kg	27.3 (1.9)	27.8 (3.8)	28.6 (2.2)^a^
Body water percentage, %	51.8 (4.2)	52.7 (4.6)	53.3 (3.8)
Estimated bone mass, kg	2.17 (0.20)	2.14 (0.25)	2.29 (0.23)^ab^
Visceral fat level	4.59 (2.22)	4.38 (2.04)	4.44 (2.57)
Basal metabolic rate, kcal/day	1100 (84)	1084 (94)	1136 (91)
Muscle mass, kg	35.8 (2.0)	35.5 (2.7)	37.2 (2.5)^ab^
Fat mass, kg	15.3 (5.4)	14.3 (5.7)	14.6 (5.1)
Body fat percentage, %	28.0 (6.5)	26.6 (7.8)	26.4 (6.3)

Values are presented as mean ± standard deviation unless otherwise indicated

^a^*P* < 0.05 for unpaired t test compared with non-consumers

^b^*P* < 0.05 for unpaired t test compared with exclusive low-fat milk consumers

On univariate analysis, the exclusive normal/high-fat milk consumers were found to have significantly higher lean body mass (mean ± standard deviation [SD], 39.4 ± 2.7 kg vs. 37.9 ± 2.2 kg and 37.6 ± 2.9 kg, *P* < 0.05) and muscle mass (mean ± SD, 37.2 ± 2.5 kg vs. 35.8 ± 2.0 kg and 35.5 ± 2.7 kg, *P* < 0.05) than the non-consumers and exclusive low-fat milk consumers (Table 2). Body water and estimated bone mass were also found to be higher in exclusive normal/high-fat milk consumers.

**Fig. 1:**
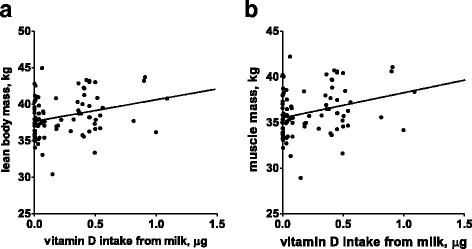
Correlation between vitamin D intake from milk and **a** lean body and **b** muscle mass. Vitamin D intake from milk was significantly correlated with (**a**) lean body mass (Pearson *r* = 0.29, 95% confidence interval (CI) 0.08–0.47, *P* < 0.01) and (**b**) muscle mass (Pearson *r* = 0.29, 95% CI 0.08–0.47, *P* < 0.01)

Speculating on the mechanism underlying the differences in lean body and muscle masses between the low-fat and normal/high-fat milk consumers, we focused on a fat-soluble and myotropic nutrient, vitamin D. The exclusive normal/high-fat milk consumers ingested more vitamin D from milk (mean ± SD, 0.49 ± 0.20 μg vs. 0.01 ± 0.02 μg, *P* < 0.0001) than the exclusive low-fat milk consumers. Furthermore, the lean body mass (Pearson *r* = 0.29, 95% confidence interval (CI) 0.08–0.47, *P* < 0.01) (Fig. 1a) and the muscle mass (Pearson *r* = 0.29, 95% CI 0.08–0.47, *P* < 0.01) (Fig. 1b) were significantly correlated with vitamin D intake from milk.

## Discussion

In the present study, we found that the consumption of normal/high-fat milk in Japanese middle-aged women was associated with higher lean body and muscle mass, which were significantly correlated with vitamin D intake from milk.

Milk is known to contain various nutrients that support myogenesis, which was exemplified in a recent study of elderly women showing that high dose of milk product intake after exercise increased thigh muscle strength [13]. In our analysis, there was a difference between normal/high-fat milk and low-fat milk in terms of the relationship between their intake and the muscle and lean body mass, which led us to focus on vitamin D, a fat-soluble and myotropic nutrient.

Previous studies have examined the association between vitamin D insufficiency and loss of muscle mass [16]. Some have shown that vitamin D deficiency was linked to sarcopenia in the elderly as well as muscle weakness, while another reported that 3 years of cholecalciferol supplementation accompanied by calcium decreased the odds of fall in elderly women by 46%, especially by 65% in the sedentary [17]. Furthermore, it was revealed that the consumption of fatty meals along with vitamin D3 supplementation significantly enhances absorption of the vitamin [18]. Taking into consideration that normal/high-fat milk contains 0.3 μg of vitamin D per 100 g of milk while low-fat milk contains almost none [19], our finding could be explained by the difference in the ingestion of the fat-soluble vitamin.

Our study has some limitations. First, the number of participants was relatively small. Second, most participants consumed milk much less frequently than their Western counterparts, in line with the report that Japanese consume an average of 120 g of milk per day, which is far less than Europeans and Americans, partly due to the difference in ethnic dietary habits [20]. Therefore, the study findings may not be generalizable to women outside of Japan. Third, the cross-sectional design of the present study does not allow for causality to be determined between body composition and milk consumption. To corroborate the observed association between normal/high-fat milk consumption and higher lean body and muscle mass, studies are warranted that enroll women with much more varied levels of milk consumption and prospectively evaluate the changes in body composition.

## Conclusions

In conclusion, normal/high-fat milk consumption is associated with higher lean body and muscle mass in Japanese women aged 40–60 years presumably due to high vitamin D intake. Normal/high-fat milk consumption could contribute to the prevention of sarcopenia in later life.

## Abbreviations

BCAA: Branched-chain amino acids
BDHQ: Brief-type self-administered diet history questionnaire
CI: Confidence interval
CVD: Cardiovascular diseases
MHR-QOL: Menopausal Health-Related Quality of Life
RXR: Retinoid X receptor
SD: Standard deviation
VDR: Vitamin D receptors
χ2: Chi Squared
